# Endoscopic vacuum therapy versus stent treatment of esophageal anastomotic leaks (ESOLEAK): study protocol for a prospective randomized phase 2 trial

**DOI:** 10.1186/s13063-021-05315-4

**Published:** 2021-06-02

**Authors:** Michael Tachezy, Seung-Hun Chon, Isabel Rieck, Marcus Kantowski, Hildegard Christ, Karl Karstens, Florian Gebauer, Tobias Goeser, Thomas Rösch, Jakob R. Izbicki, Christiane J. Bruns

**Affiliations:** 1 Department of General, Visceral and Thoracic Surgery, University Medical Center Hamburg-Eppendorf, Hamburg, Germany; 2 Department of General, Visceral, Cancer and Transplant Surgery, University Hospital Cologne, Kerpener Str. 62, 50937 Cologne, Germany; 3 Department of Gastroenterology and Hepatology, University Hospital Cologne, Cologne, Germany; 4 Department of Interdisciplinary Endoscopy, University Medical Center Hamburg-Eppendorf, Hamburg, Germany; 5 Institute of Medical Statistics and Bioinformatics, University of Cologne, Cologne, Germany

**Keywords:** Self-expanding metal stents, Endoscopic vacuum therapy, Anastomotic leaks, Esophagectomy, Esophageal cancer, Interventional endoscopy

## Abstract

**Background:**

Intrathoracic anastomotic leaks represent a major complication after Ivor Lewis esophagectomy. There are two promising endoscopic treatment strategies in the case of leaks: the placement of self-expanding metal stents (SEMS) or endoscopic vacuum therapy (EVT). Up to date, there is no prospective data concerning the optimal endoscopic treatment strategy. This is a protocol description for the ESOLEAK trial, which is a first small phase 2 randomized trial evaluating the quality of life after treatment of anastomotic leaks by either SEMS placement or EVT.

**Methods:**

This phase 2 randomized trial will be conducted at two German tertiary medical centers and include a total of 40 patients within 2 years. Adult patients with histologically confirmed esophageal cancer, who have undergone Ivor Lewis esophagectomy and show an esophagogastric anastomotic leak on endoscopy or present with typical clinical signs linked to an anastomotic leak, will be included in our study taking into consideration the exclusion criteria. After endoscopic verification of the anastomotic leak, patients will be randomized in a 1:1 ratio into two treatment groups. The intervention group will receive EVT whereas the control group will be treated with SEMS. The primary endpoint of this study is the subjective quality of life assessed by the patient using a systematic and validated questionnaire (EORTC QLQ C30, EORTC QLQ-OES18 questionnaire). Important secondary endpoints are healing rate, period of hospitalization, treatment-related complications, and overall mortality.

**Discussion:**

The latest meta-analysis comparing implantation of SEMS with EVT in the treatment of esophageal anastomotic leaks suggested a higher success rate for EVT. The ESOLEAK trial is the first study comparing both treatments in a prospective manner. The aim of the trial is to find suitable endpoints for the treatment of anastomotic leaks as well as to enable an adequate sample size calculation and evaluate the feasibility of future interventional trials. Due to the exploratory design of this pilot study, the sample size is too small to answer the question, whether EVT or SEMS implantation represents the superior treatment strategy.

**Trial registration:**

ClinicalTrials.gov NCT03962244. Registered on May 23, 2019.

DRKS-ID DRKS00007941

**Supplementary Information:**

The online version contains supplementary material available at 10.1186/s13063-021-05315-4.

## Background

Intrathoracic anastomotic leaks occur in up to 20% after Ivor Lewis esophagectomy. This complication is associated with a prolonged hospital stay and increased postoperative mortality and therefore one of the most feared complications in visceral surgery [1–4]. During the last years, there has been a controversial debate about the optimal treatment strategy of anastomotic leaks ranging from conservative over interventional endoscopic to surgical approaches [4–6]. While early anastomotic leaks, particularly in the presence of sepsis, as well as extended conduit necrosis still remain a recommended indication for surgical revision, growing expertise suggests endoscopic treatment in the case of leaks, which are defined as localized retentions connected to the site of anastomotic insufficiency [7–10]. The current gold standard in the endoscopic management of anastomotic leaks seems to be the usage of self-expanding metal stents (SEMS), with success rates of approximately 70–81%. Endoscopic vacuum therapy (EVT) represents a newer alternative that has been introduced by Wedemeyer and colleagues in 2008. Since then, an increasing number of patients has been treated with this method showing promising results in retrospective studies: The healing rates range between 67 and 100% [11–18]. Both treatment strategies present advantages and drawbacks: While SEMS provide a complete seal of the leak and maintain the esophageal passage for oral intake, they might induce ischemia and require the placement of additional drainages in some of the patients. Stent migration is a common problem calling for re-interventions. Endoscopic vacuum therapy offers continuous fluid collection facilitating the granulation process and reducing bacterial proliferation, but needs to be changed every 2–5 days, requires a trans-nasal suction drainage, and forbids oral nutrition [19, 20].

A recent systematic review identified five retrospective studies comparing both methods and the meta-analysis of the data suggested that EVT had a higher rate of leakage closure than SEMS [21]. Nevertheless, no clear recommendations can be made due to methodological weaknesses of the underlying retrospective studies. So far, there is no prospective data concerning the optimal endoscopic treatment for anastomotic leaks calling for comparative studies to strengthen the evidence. To our knowledge, the ESOLEAK trial is the first study to compare SEMS and EVT for the treatment of anastomotic leaks after esophagectomy in a prospective, randomized design.

## Methods

### Aim and design of the study

The aim of the open, randomized ESOLEAK study is to investigate in a prospective manner two different endoscopic treatment modalities of anastomotic leakages after Ivor Lewis esophagectomy, namely the endoscopic placement of self-expanding metal stents (SEMS) and the endoscopic vacuum therapy (EVT).

**Fig. 1:**
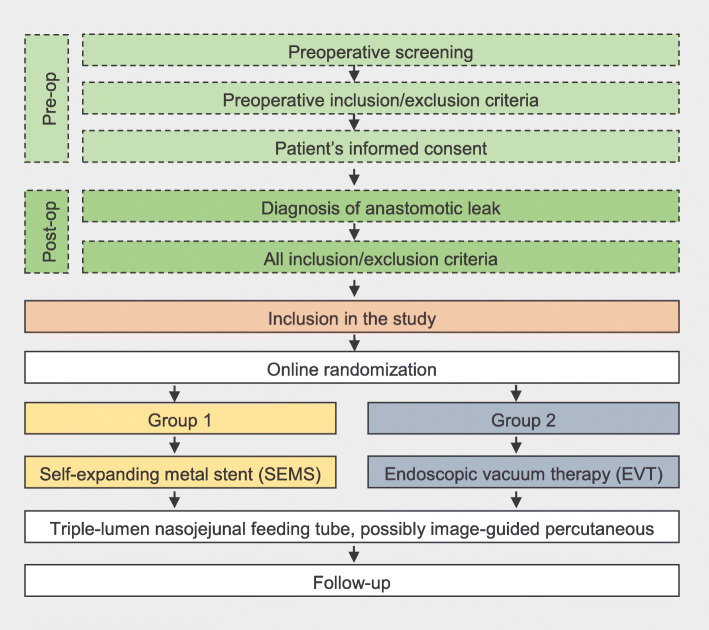
Flow chart illustrating the study design

The trial will be conducted as a phase 2 randomized trial at two tertiary medical centers with the aim to define appropriate outcome measures and study population size for a larger interventional trial. The study design is illustrated in Fig. 1.

### Patients

This study will be conducted at the University Hospital of Cologne and the University Medical Center Hamburg-Eppendorf and include a total of 40 patients (n=40). The number of patients is estimated in order to show the feasibility of the trial protocol and to provide data to calculate the sample size for a larger randomized prospective interventional trial.

All patients with resectable esophageal cancer will be screened for eligibility to be enrolled in the study. Informed consent will be obtained preoperatively before the inclusion criterion of an anastomotic leak is satisfied.

#### Inclusion criteria

1. Histologically confirmed esophageal cancer or similarly operated neoplasia
2. Esophagectomy with intrathoracic esophagogastric anastomosis
3. Endoscopically diagnosed esophagogastric anastomotic leak
4. Clinical signs or symptoms due to the leak or elevated inflammatory parameters, most likely linked to the anastomotic leak
5. ≥ 18 years of age
6. Patient’s ability to understand the study extent and consequences
7. Signed informed consent form

#### Exclusion criteria

1. Macroscopically incomplete resection of the tumor (R2), palliative resection
2. Endoscopically verified necrosis or critical ischemia of the conduit
3. Size of the leak larger than 50% of the anastomotic circumference
4. Impossibility to place a CT- or ultrasound-guided drainage if evacuation is needed in case of SEMS placement
5. Early (≤ 48 h postoperative) and late (> 4 weeks postoperative) anastomotic leaks
6. Therapeutic anticoagulation
7. Signs of severe sepsis requiring urgent surgical treatment
8. Pregnancy and lactation

### Randomization

In case of clinical, radiological, or laboratory suspicion of an anastomotic leak after Ivor Lewis esophagectomy, a diagnostic upper gastrointestinal endoscopy will be performed. If an anastomotic leak is verified endoscopically and all inclusion and exclusion criteria are met, patients will be randomly assigned to either control (SEMS) or experimental (EVT) group with a 1:1 allocation using a web-based, centralized permuted block randomization system. The allocation will be final and the researchers will not be able to influence the results. Due to the character of the intervention, a blinding is impossible.

### Trial intervention

#### Endoscopy

**Fig. 2:**
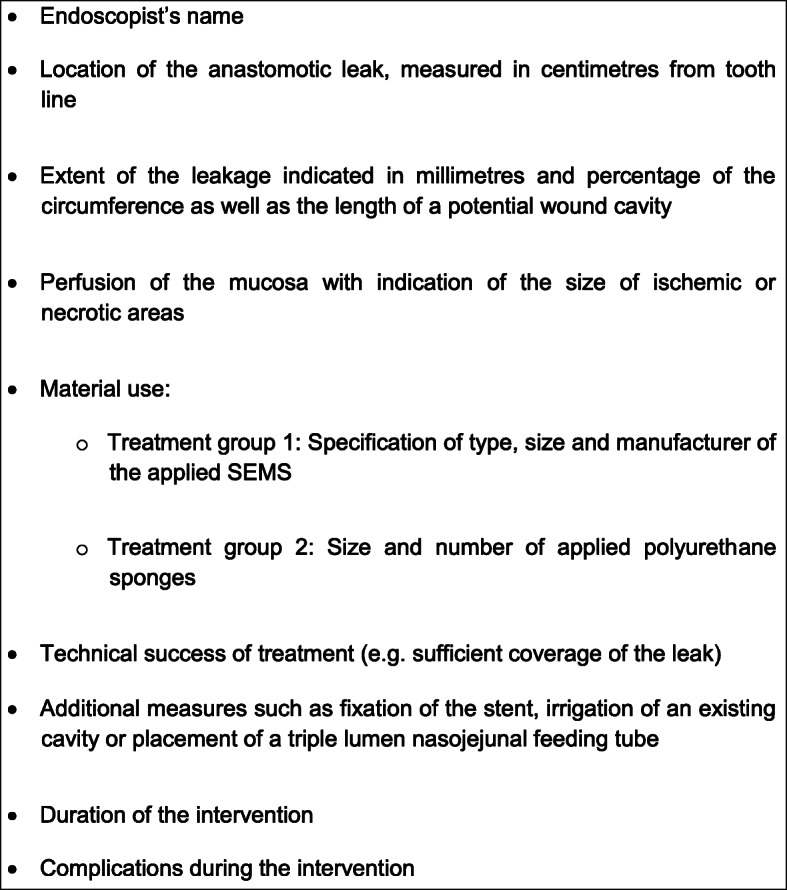
Standardized documentation of diagnostic and therapeutic endoscopies

Each diagnostic endoscopy in the course of the study will be performed by a maximum of three experienced endoscopists per center following a standardized protocol and will be described in detail and visually documented in photo and video (Fig. 2). Regardless of the group allocation, irrigation should only be performed in endoscopically accessible cavities. After the allocation to one of the groups, we ensure endoscopic treatment within a period of 12 h after diagnosis by implementing a 24/7 on-call service.

#### Intervention group (EVT)

Before the endoscopic placement of the vacuum therapy system, a triple lumen nasojejunal feeding tube is inserted for gastric decompression and to ensure intestinal feeding throughout the duration of the EVT.

The sponge system can be either positioned inside the esophageal lumen (endoluminal position) or into the extraluminal cavity adjacent to the anastomotic leak (extraluminal position). Anastomotic leaks without an accessible extraluminal cavity will be treated by endoluminal sponge placement whereas anastomotic leaks with larger cavities are treated by inserting a manually customized sponge into the cavity. The sponge system will be placed using an overtube and a pusher. Hereafter, the sponge position will be controlled endoscopically and may be adjusted using a forceps. Then the drainage tube is placed via the nasal cavity and secured with adhesive strips or a nasal tube retaining system (e.g., AMT Bridle™, Applied Medical Technology, USA). Subsequently, the drainage tube will be connected to an electronic vacuum pump with a defined continuous suction (e.g., VivanoTec®, Hartmann AG, Germany). Since there is no data concerning the selection of the ideal suction power for EVT, we decided to use a suction power of 125 mmHg, which we have established as a standard in our center in Cologne and Hamburg since 2010. The system exchange or removal is scheduled 3–5 days after the initial placement. To ensure an atraumatic removal of the sponge system, the drainage tube is disconnected from the vacuum pump and 40 ml of saline solution is injected into the tube to wet the sponge and thus facilitate removal. The EVT may be terminated either after full closure of the leak or complete lining of the cavity with granulation tissue. Dislocation or migration of the sponge system is classified as complications.

#### Control group (SEMS)

Stents used in this study must be self-expanding, partially covered metal stents (CE certified - CE0197). The product will be narrowed down to a few models, but the final decision regarding the stent type and size will be left up to the endoscopist. The stent may be secured in its position by using clips or endoscopic suturing techniques. To achieve optimal coverage and seal of the leak, the flare ends of the stent need to be proximal and distal to the leak. In case of stent dislocation or migration, the stent may be repositioned and fixed or replaced by another model or size with better adjustment. This scenario will be documented as a complication. After successful stent placement, a triple lumen nasojejunal feeding tube suitable for intestinal feeding and gastric decompression is inserted. The stent will be removed or replaced after 21 days (±2 days).

#### Image-guided drainage insertion

Regardless of the group allocation, each patient receives a CT scan of the thorax and abdomen, which should preferably be performed prior to study treatment. Oral and intravenous CT contrast is used to diagnose possible fluid collections. In case of insufficiently drained fluid collections, an ultrasound- or CT-guided drainage will be inserted by a radiologist.

#### Supportive therapy

All patients receive supportive therapy such as transfusions of red cell concentrates or analgesia according to international intensive care standards and guidelines. Regardless of the group allocation, an enteral nutrition regimen is implemented via the jejunal lumen whereas continuous drainage of reflux with a suction of 10mmHg is ensured via the gastric lumen. Immediately after diagnosis of an anastomotic leak, all patients receive a calculated broad-spectrum anti-infective therapy and close monitoring, if necessary by intensive care. All intensive care treatments, including organ replacement therapies, may be applied as required.

### Follow-up

Following the primary endoscopic intervention, the patient will be treated according to the clinic’s standard operating procedures. If the patient’s clinical status does not improve within 72 h, endoscopic reevaluation and/or another CT scan will be performed. In case of new fluid collections, a drainage will be inserted. If endoscopy shows necrosis or severe ischemia of the interponate or a significant increase of the anastomotic leak size, a surgical revision will be evaluated by the treating physician taking into account the patient’s clinical status.

During the hospital stay, the following data are determined on days 3, 7, 14, 21, and 28 and then weekly (±1 day) after the diagnosis of anastomotic leakage as illustrated in Table 1: vital signs, SOFA score, duration of ventilation, leukocytes, CRP, and procalcitonin value, if applicable [22]. All relevant endoscopic, surgical, and non-surgical complications throughout the hospital stay including postoperative death will be documented in the CRF (Table 1). Follow-up examinations are performed 3 and 6 months (±14 days) after inclusion (Table 1). In addition to assessing the quality of life and the clinical status, the following details are documented on follow-up visits: body weight, Karnofsky index, presence of dysphagia or a symptomatic stenosis, oncologic status (relapse/remission), and tumor-specific therapy, if applicable. The recurrence rate and mortality including the cause of death will be documented beyond the last study visit contacting the treating physician or the patient.

**Table 1:** Trial events according to treatment and follow-up plan

		Intervention phase		Follow-up (time after informed consent)		
Trial checkpoint	Screening	Prior to treatment	Hospital stay after inclusion	3 months (± 14 days)	6 months (± 14 days)	12 months (± 14 days)
**Informed consent**	X					
**Inclusion/exclusion criteria**	X					
**Medical history, physical examination, vital signs**	X^a^	X	X^b^	X	X	X
**Diagnostic upper GI endoscopy**		X				
**CT scan**		X	(X)		x	
**ECG**	X					
**Laboratory tests**	X	X	X^b^	X	X	X
**Operative report**		X	X			
**Postoperative complications, treatment failure**		X	X			
**SOFA score**		X	X^b^			
**QoL questionnaire**	X		X^c^	X	X	X
**Treatment costs**			X			
**Monitoring of SAE**		X (from randomization to discharge from hospital + 30 days)				
**Overall survival**		X				

^a^At inclusion with detailed medical history and extensive physical exam, otherwise only vital signs, weight, and Karnofsky index

^b^On days 3, 7, 14, and 21 (±1 day)

^c^On day 10 and prior to discharge from hospital

To reduce the rate of loss of follow-up, we chose short-term aspects as primary outcome parameters and planned the follow-up visits according to the routinely conducted quarterly oncologic follow-up exams in each center.

### Data collection

#### Patient characteristics

All preoperative screening parameters, such as patient’s history (smoking status, alcohol intake, presence of diabetes, and previous thoraco-abdominal operations), clinical status (body height, body weight, blood pressure, heart rate, activity index, Karnofsky index, and ASA score), and tumor-specific parameters (histological confirmation of esophageal cancer, preoperative TNM and RECIST classification, results of clinical staging exams and tests conducted according to the national guideline for esophageal cancer (German S3 Guideline, 2018) and previous tumor-specific treatment), as well as all inclusion and exclusion criteria, will post hoc be included in the CRF.

#### Feasibility of a RCT

To determine the feasibility of a RCT, screening and recruitment logs are used to evaluate the recruitment and retention rate. Moreover, the number of patients not fulfilling the inclusion and exclusion criteria is documented to evaluate the suitability of the parameters.

### Effect evaluation

#### Primary effect measure

The primary endpoint of this study is the subjective quality of life assessed by the patient using a systematic and validated questionnaire from the European Organisation for the Research and Treatment of Cancer (EORTC QLQ C30, EORTC QLQ-OES18 questionnaire), which incorporates nine multi-item scales: five functional scales (physical, role, cognitive, emotional, and social), three symptom scales (fatigue, pain, and nausea and vomiting), and a global health and quality-of-life scale [23–25]. The evaluation will be performed at screening, after randomization before treatment, day 7 (±2) after randomization, upon discharge from the hospital, and 1 (±5 days), 3, and 6 months after randomization (±14 days). After inclusion, data will be transferred to the CRF.

#### Secondary effect measures

1. Cure rate of patients within 44 days postoperatively. Cure is defined as follows:
  ○ No leak can be detected clinically, endoscopically, and/or radiologically *or*
  ○ Patients are able to be discharged from the hospital despite persistent leakage (treated with SEMS or drainages), once they are in a clinically stable state without signs of acute inflammation and tolerate oral food intake.
  ○ In case of death within 44 days postoperatively and persistent leak, patients are classified as “not cured.”
2. Period of hospitalization, defined as the time in days from the day of surgery (day 0) until discharge from the hospital to ambulant or for follow-up treatment (except early rehabilitation or neurological rehabilitation)
3. Rate of and reasons for treatment failure, defined as a necessary change of the treatment strategy or postoperative deaths, that are directly or indirectly associated with the anastomotic leak
4. Rate and type of surgical revisions with indication of the reasons
5. General complications (surgical and non-surgical complications) and treatment-specific complications such as stent dislocations, which will be classified according to the Clavien-Dindo classification and the Comprehensive Complication Index [22]
6. Mortality, defined as postoperative death within 30 and 90 days (30-day and 90-day mortality) and during the hospitalization period. In addition, we determined whether the patients died as a direct or indirect result of the insufficiency.
7. Duration of intensive care treatment (starting from diagnosis of the anastomotic leak)
8. Influence of treatment on possible sepsis, as measured by the SOFA score [26, 27]
9. Ventilation time (measured ventilation time in hours from inclusion to extubation or continuously spontaneously breathing tracheostomized patients, respectively)
10. Duration of therapy (from the time of inclusion until the removal of the last drainage or stent)
11. Number of endoscopies throughout the trial with indication of number of SEMS or vacuum system replacements and/or revisions
12. Duration of healing (from the day of inclusion until no leakage can be detected endoscopically)
13. Time from inclusion to oral food intake
14. Treatment costs (inpatient and outpatient)
15. Occurrence of symptomatic stenosis with indication of chosen specific treatment

Secondary outcome measures will be registered up to 1 year after inclusion.

During each intervention and every study visit during the complete course of the intervention and follow-up, specific complications potentially related to the intervention are documented. AEs and SAEs are collected, and relations to the study intervention interpreted and compared between the two groups and the results published.

#### Publication policy

Trial results will be published in a medical journal and released to the participating physicians, patients, and general medical community. Full trial protocol, full study report, anonymized results of the trial, and statistical data set will be provided on demand to the medical community.

### Duration of study and sample size calculation

**Fig. 3:**
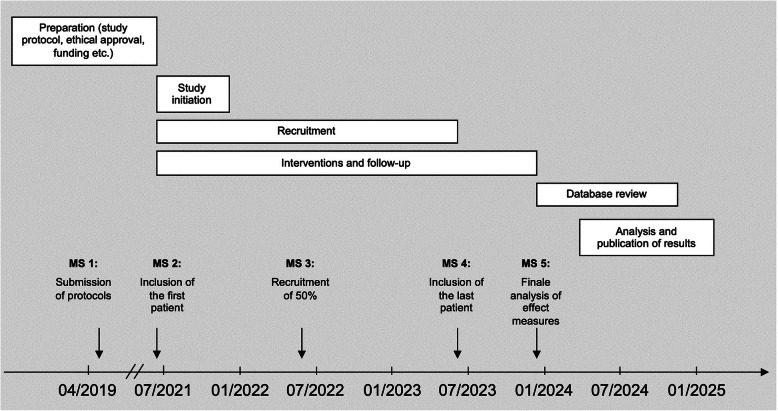
Timeline and important milestones of the study

Due to insufficient and reliable data regarding suitable endpoints for the treatment of anastomotic leaks, a sample size calculation is not possible at this point. The aim of this study is, among other things, to evaluate the feasibility of a RCT and to collect data in order to enable adequate sample size calculation for future larger interventional trials. Based on the rate of leakage and an estimated inclusion rate of 80% of the patients, the duration of the study is calculated to be 48 months with a 24-month recruitment phase (Fig. 3).

#### Trial management and monitoring

Trial management is conducted by the principal investigator and research physicians of each participating study center who are part of the trial management committee. Standardized monitoring of 20% of the data and essential material is conducted by an external clinical research organization. Both therapeutic regimens applied in this are clinically well-established and investigated therapies and there will be no blinding. Therefore, no study-specific harm was identified and the study was classified as low-risk. As a consequence, no DMC is planned.

### Statistical analysis

The primary analysis and the analysis of the secondary endpoints will be conducted according to the intention-to-treat (ITT) principle including all patients. Each patient will be analyzed independent of the group allocation.

To assess the quality of life, either a t-test for independent groups or a Mann-Whitney U test is performed at the two-sided level of 5% depending on the distribution type.

For statistical evaluation of the secondary endpoints, parametric and non-parametric analyses will be conducted depending on the distribution type.

*p*-values < 0.05 are considered statistically relevant, but merely in a descriptive manner. Quantitative variables are described by mean value, standard deviation, and quartiles, and qualitative variables by absolute and relative frequency. The effect size is calculated as follows: With two unrelated samples (*n* = 20), α = 0.05, and power = 0.8, an effect of 0.909 can be calculated.

All comparisons with respect to the targets explicitly mentioned elsewhere are exploratory and therefore differences are considered significant if the respective p-value is less than 0.05. Subgroup analyses regarding, e.g., SOFA scores are planned.

The analysis of the data will be performed as an intention-to-treat analysis. In case of missing data, the effect on the results will be assessed via sensitivity analysis of augmented data sets. Reasons for missing data will be analyzed and qualitatively compared between the two groups.

In advance, all planned analyses will be specified in a statistical analysis plan (SAP), which will be finalized based on pooled data analysis before the first comparative analysis. No interim analysis is planned for this trial.

## Discussion

Anastomotic leaks after esophagectomy remain a major postoperative complication, representing a potentially life-threatening condition for the patient. Thus, new therapeutic approaches need to be well established and compared to the current standard to achieve the best possible outcome. Up to date, there has been more of an eminence-based rather than an evidence-based therapeutic approach to anastomotic leaks justified by the proclamation of the importance of individualized treatment strategies. Treatment of anastomotic leaks is already difficult and complicated by the lack of defined criteria such as the size of the leak or the existence of a wound cavity for the choice of the best endoscopic treatment strategy. So far, this calls for an individualized treatment regime, which is chosen depending on the expertise and equipment of the treating clinic. In our opinion, this is not the optimal basis for such an important and consequential therapeutic decision, so that the long-term goal should at least be the attempt to establish a standardized treatment. With our study, we would like to lay a first foundation to possibly be able to answer the question in the future, whether a standardized protocol can be established for such a complex clinical situation as an anastomotic leak. This also implies taking into account some possible protocol deviations.

Current evidence is not conclusive enough to make clear recommendations as well as to define variables that favor one treatment strategy over the other. The latest meta-analysis comparing implantation of SEMS with EVT suggested a higher success rate for EVT and thus calling for further prospective interventional trials [21]. However, the underlying retrospective trials do not permit a final recommendation due to significant bias and methological weaknesses. Prospective data regarding this important question are missing.

The ESOLEAK trial is the first prospective study comparing both treatments in the presence of esophageal anastomotic leaks. Due to the exploratory design of this pilot study, obviously, the sample size is too small to answer the question, whether EVT or SEMS implantation represents the superior treatment strategy. The aim of the trial is to evaluate the feasibility of planned RCTs and to find suitable endpoints for the treatment of anastomotic leaks as well as to enable an adequate sample size calculation for future interventional trials. It is very difficult to select an endpoint that does equal justice to both therapeutic approaches taking into account their different ways of application: continuous therapy with few endoscopies (SEMS) vs. frequent endoscopic change and control (EVT). By selecting the quality of life as a primary endpoint, we chose a soft, but from our perspective very important and often underrepresented parameter that is appropriate to analyze patients in both treatment arms in a standardized manner.

In summary, the two main endoscopic strategies for contained leaks after Ivor Lewis esophagectomy—SEMS and EVT—are compared in a prospective, randomized fashion for the first time. The results of the ESOLEAK study are intended to lay the foundation for larger interventional trials tackling this issue and to contribute essential evidence to the treatment of this severe complication.

## Trial status

Study protocol version 1.1, 23.05.2019

Estimated study start date: 1 December 2020

Approximate date when recruitment will be completed: 1 December 2022

Not yet recruiting

## Supplementary Information

**Additional file 1.** WHO Trial Items

**Additional file 2.** German Study Register

## Data Availability

The data of the current study is not planned to be used in further trials.

## Abbreviations

ASA score: American Society of Anesthesiologists score
CE certified: Conformité Européenne
CRF: Case report form
CRP: C-reactive protein
ECG: Electrocardiography
EORTC QLQ: European Organization for Research and Treatment of Cancer Quality of Life Questionnaire
EVT: Endoscopic vacuum therapy
ITT: Intention-to-treat
ml: Milliliters
mmHg: Millimeters of mercury
SAE: Serious adverse event
SAP: Statistical analysis plan
SEMS: Self-expanding metal stents
SOFA score: Sequential Organ Failure Assessment score
RCT: Randomized controlled trial
RECIST: Response Evaluation Criteria in Solid Tumors
TNM: Classification of Malignant Tumors
