# Supplementary material for: Endoscopic vacuum therapy versus stent treatment of esophageal anastomotic leaks (ESOLEAK): study protocol for a prospective randomized phase 2 trial
Source: Trials. 2021 Jun 2;22:377. doi: 10.1186/s13063-021-05315-4 (PMC8170795; doi:10.1186/s13063-021-05315-4)
Supplement: Supplementary file 1 — Additional file 1. WHO Trial Items [file 13063_2021_5315_MOESM1_ESM.pdf]

World Health Organization

International Clinical Trials Registry Platform Search Portal

Main

Note: This record shows only 22 elements of the WHO Trial Registration Data Set. To view changes that have been made to the source record, or for additional information about this trial, click on the URL below to go to the source record in the primary register.

**Register:**	ClinicalTrials.gov
**Last refreshed on:**	6 April 2020
**Main ID:**	NCT03962244
**Date of registration:**	21/05/2019
**Prospective Registration:**	Yes
**Primary sponsor:**	University Hospital of Cologne
**Public title:**	Stent Therapy Versus Endoscopic Vacuum Therapy for Anastomotic Leaks After Esophagectomy EsoLeak
**Scientific title:**	Endoscopic Management of ESOphago-gastric Anastomotic LEAKages (EsoLeak): Stent Therapy Versus Endoscopic Vacuum Therapy
**Date of first enrolment:**	April 1, 2020
**Target sample size:**	40
**Recruitment status:**	Not yet recruiting
**URL:**	https://clinicaltrials.gov/show/NCT03962244
**Study type:**	Observational
**Study design:**	
**Phase:**	

Countries of recruitment

Germany

Contacts

Name: Seung-Hun Chon, MD
Address:
Telephone: +49221478 4864
Email: seung-hun.chon@uk-koeln.de
Affiliation:

Name: Michael Tachezy, MD
Address:
Telephone:
Email:
Affiliation: Universitätsklinikum Hamburg-Eppendorf (UKE)

Name: Seung-Hun Chon, MD
Address:
Telephone:
Email:
Affiliation: University Hospital of Cologne

Key inclusion & exclusion criteria

Inclusion Criteria:

- Histologically confirmed oesophageal carcinoma or similarly operated neoplasia (e.g., GIST, NET, subepithelial tumors)

- Esophagectomy with an intrathoracic esophago-gastric anastomosis

- Radiologically or endoscopically diagnosed esophago-gastric anastomotic leakage

- Clinical symptoms / symptoms due to insufficiency or increase in signs of inflammation, most likely as a result of anastomotic leakage

- Age =18 years

- To empower the patient to understand the scope of the study and its consequences or

information, to consent to it and to sign the educational documents.

- Written declaration of consent of the patient to be included. If the patient is unable to sign by hand, a witness must confirm the oral examination by signature.

Exclusion Criteria:

- Macroscopically incompletely resected tumor (R2), palliative resection

- Endoscopically verified necrosis or critical ischemia of the anastomotic region of the interponate

- Size of insufficiency more than 50% of circumference

- Impossibility of radiological interventional insertion of a drainage

- Early anastomotic leak (= 48 hours postoperatively), late insufficiencies (> 4 weeks)

- Therapeutic anticoagulation

- Severe septic shock that indicates surgical therapy

- Pregnant and lactating women

Age minimum: 18 Years
Age maximum: N/A
Gender: All

Health Condition(s) or Problem(s) studied

Leaks, Anastomotic

Intervention(s)

Device: EsoSponge

Device: Self-Expanding Metal Stent

Primary Outcome(s)

Satisfaction of treatment assessed by EORTC QLQ - OES18 [Time Frame: 6 months]

Secondary Outcome(s)

Secondary ID(s)

19-1201

Source(s) of Monetary Support

Please refer to primary and secondary sponsors

Secondary Sponsor(s)

Universitätsklinikum Hamburg-Eppendorf

Ethics review

Results

Results available:
Date Posted:
Date Completed:
URL:

Disclaimer: Trials posted on this search portal are not endorsed by WHO, but are provided as a service to our users. In no event shall the World Health Organization be liable for any damages arising from the use of the information linked to in this section. None of the information obtained through use of the search portal should in any way be used in clinical care without consulting a physician or licensed health professional. WHO is not responsible for the accuracy, completeness and/or use made of the content displayed for any trial record.

Copyright - World Health Organization - Version 3.6 - Version history
