# Supplementary material for: Endoscopic vacuum therapy versus stent treatment of esophageal anastomotic leaks (ESOLEAK): study protocol for a prospective randomized phase 2 trial
Source: Trials. 2021 Jun 2;22:377. doi: 10.1186/s13063-021-05315-4 (PMC8170795; doi:10.1186/s13063-021-05315-4)
Supplement: Supplementary file 2 — Additional file 2. German Study Register [file 13063_2021_5315_MOESM2_ESM.pdf]

Deutsches Register Klinischer Studien
German Clinical Trials Register

Studienbeschreibung

Titel der Studie

Endoskopische Stentversorgung versus konservative Behandlung von ösophago-enterischen Anastomoseninsuffizienzen

Studienakronym

ESOLEAK

Internetseite der Studie

http://na

Allgemeinverständliche Kurzbeschreibung

Die einzig kurative Behandlungsmöglichkeit bei bösartigen Speiseröhren- und Magentumoren ist die operative Entfernung, wenn noch keine Absiedelungen in andere Organe aufgetreten sind. Eine der häufigsten Komplikationen ist eine Leckage der neu geschaffenen Verbindung zwischen Speiseröhre und Magen bzw. Darm. Diese stellt aufgrund der teilweise schwerwiegenden Folgekomplikationen eine der gefürchtetsten Komplikationen in der Chirurgie dar. Die Sterblichkeit ist, trotz stetiger Verbesserungen der operativen und postoperativen Behandlung, weiterhin sehr hoch (bis zu 50%). Die optimale Behandlungsstrategie dieser Komplikation ist umstritten. Das Spektrum der Behandlungsmöglichkeiten reicht von konservativen über endoskopischen Behandlungen bis hin zu aggressivem chirurgischen Vorgehen. Eine systematische Übersichtarbeit unserer Arbeitsgruppe hat ergeben, dass aufgrund des Fehlens von Studien mit hohem Evidenzgrades eine Überlegenheit der viel beschriebenen und durchgeführten endoskopischen Behandlung der konservativen Behandlung nicht belegt werden kann ist. Auch die Daten aus unserer Klinik bestätigen dieses Ergebnis.
Die geplante Studie soll untersuchen, ob die endoskopische Therapie mittels Stent-Einlage der konservativen Therapie tatsächlich überlegen ist. Beides stellen Standardtherapien dieser Komplikation dar. Es wird angenommen, dass durch die Stent-Einlage die Rate geheilter Patienten bzw aus dem Krankenhaus entlassener Patienten am 44. Tag von 50% auf 80% erhöht werden kann. Daraus ergibt sich eine Fallzahlkalkulation von insgesamt 78 Patienten, die per Zufall in die Behandlungsarme zugewiesen werden. Des Weiteren werden zahlreiche andere Endpunkte untersucht, wie die Sterblichkeit, die Rate an Komplikationen, die Lebensqualität, die Behandlungskosten u.v.m.

Wissenschaftliche Kurzbeschreibung

Die ösophago-enterischen Anastomoseninsuffizienzen stellen, aufgrund der hohen Rate an Folgekomplikationen mit intensivmedizinischer Behandlung, langer Krankenhausverweildauer und signifikanter Mortalität eine der gefürchtetsten Komplikationen der Viszeralchirurgie dar. Durch Optimierungen der chirurgischen Techniken und des peri-operativen konnte in den letzten Jahrzehnten die Mortalitätsrate deutlich gesenkt werden. Während die Therapie von Insuffizienzen der zervikal gelegenen Anastomosen üblicherweise aus einer einfachen Drainage durch Eröffnung der Wunde besteht, ist die optimale Behandlungsstrategie bei

den intrathorakalen und abdominellen Insuffizienzen weitestgehend umstritten und zeigt eine weite Bandbreite von konservativen bis hin zu radikal chirurgischen Behandlungen.

Chirurgische Revisionen mit Reparatur oder Neuanlage der Anastomose oder einer Resektion des Interponates sind deswegen nur bei ausgedehnten Nekrosen und bei sehr frühen Leckagen indiziert. In den letzten Jahren kamen nicht-chirurgische, radiologisch-interventionelle und endoskopische Behandlungsmöglichkeiten hinzu, die bessere Ergebnissen bei reduzierter Sterblichkeit für sich reklamierten, mit jeweils unterschiedlichen publizierten Behandlungsalgorithmen. Die Evidenz für diese Therapien ist bei nahezu ausschließlichen retrospektiven Fallserien oder Fall-Kontroll-Studien sowie großer Heterogenität in Patientenzusammensetzung und Detailmanagement allerdings niedrig; zudem wurden oft iatrogene Perforationen und Anastomosenkomplikationen gemeinsam ausgewertet.

Eine eigene Übersicht über 92 Studien mit etwa 1200 Patienten bestätigte die Heterogenität der Fallserien; man kann die Behandlungen im Wesentlichen in drei Gruppen einteilen, konservative Behandlungen einschliesslich nicht-chirurgischer Drainageeinlage, endoskopischen Interventionen (Fibrin-Kleber, Clipping, Tubus und Stents) und die operative Revision. Während die operative Revision der Anastomose weitestgehend als back-up Behandlung beschrieben wird, werden für die konservativen und endoskopischen Therapien verschiedene Indikationen beschrieben. In der gepolten Analyse zeigte sich eine Überlegenheit der endoskopischen und/oder konservativen gegenüber der operativen Leckage-Therapie bezüglich der Sterblichkeit (31.4%), aber keine klaren Unterschieden zwischen den beiden nicht-chirurgischen Therapieformen (12.3% und 10.7% Mortalität). Unter der Berücksichtigung des geringen Evidenzlevels der zugrunde liegenden Studien, können nur sehr vorsichtige Schlüsse gezogen werden: Die endoskopische und konservative Behandlung scheint der operativen Revision bezüglich der Sterblichkeit überlegen zu sein, es zeigte sich aber keine Überlegenheit der endoskopischen Behandlungen gegenüber den konservativen. Welche die optimale endoskopische Therapie darstellt, kann nicht gefolgert werden anhand der bisher publizierten Daten.

In der Aufarbeitung unserer eigenen Daten wurden insgesamt 87 Patienten identifiziert, die entweder primär konservativ (beinhaltet u.a. eine Drainageeinlage bei Verhalt- und Trilumensonden-Einlage zur enteralen Ernährung; n=39), endoskopisch mittels Stent, Tubus oder Sponge (n=34) oder mittels operativer Revision behandelt wurden (n=13). Die Gruppe der konservativ und endoskopisch behandelten Patienten war hinsichtlich der Vorerkrankungen und der bei Diagnose auftretenden Ko-Morbiditäten nicht unterschiedlich. Aufgrund einer Änderung der Behandlungsabläufe im Jahre 2009, wurden initial keine endoskopischen Behandlungen mehr durchgeführt. Es zeigten sich keine signifikanten Unterschiede bezüglich der Krankenhaussterblichkeit und 30 Tage-Mortalität oder auch der Krankenhaus- und Intensivstationsverweildauern der Patienten. Auch der perorale Kostaufbau wurde nach endoskopischer Therapie nicht signifikant früher begonnen.

Da unter den verschiedenen endoskopischen Methoden die endoskopische Einlage eines selbst-expandieren Stents die häufigsten angewandte zu sein scheint, soll die vorliegende prospektiv-randomisierte Studie dieses mit dem konservativen Vorgehen vergleichen. Wichtig sind zudem in beiden Gruppen die Notwendigkeit einer radiologisch-interventionellen (CT- oder sonographisch- gesteuerten)

Drainage-Einlage und die Einlage einer Ernährungssonde zur enteralen Ernährung.

Organisatorische Daten

- DRKS-ID der Studie: **DRKS00007941**
- Registrierungsdatum im DRKS: **14.04.2015**
- Registrierungsdatum im Partnerregister oder anderem Primärregister: **[---]***
- Wissenschaftsinitiierte Studie (IST/IIT): **ja**
- Votum der Ethikkommission: **Positives Votum/Zustimmende Bewertung**
- (federführende) Ethikkommissions Vorlage-Nr.: **PV4905 , Ethik-Kommission der Ärztekammer Hamburg**

Sekundäre IDs

Untersuchte Krankheit/Gesundheitsproblem

- ICD10: **C15 - Bösartige Neubildung des Ösophagus**
- ICD10: **C16 - Bösartige Neubildung des Magens**

Interventionsgruppen/Beobachtungsgruppen

- Arm 1: **Im Falle einer Anastomoseninsuffizienz einer Ösophago-enterischen Anastomose nach erweiterter Gastrektomie oder Ösophagektomie wird endoskopisch ein selbst-expandierender Stent (Metall oder Plastik) über die Anastomoseninsuffizienz eingebracht. Wenn notwendig, erfolgt die Einlage einer Ernährungssonde und eine Drainage im Falle eines nicht drainierten Verhalten sowie das gesamte erforderliche Spektrum der konservativen und intensivmedizinischen Behandlungsformen.**
- Arm 2: **Kontrollgruppe. Es wird primär keine endoskopische Therapie durchgeführt, lediglich eine Trilumensonde und im Falle eines Verhaltes eine Drainage eingebracht inklusive des gesamten erforderlichen Spektrums der konservativen und intensivmedizinischen Behandlungsformen.**

Charakteristika

- Studientyp: **Interventionell**
- Studientyp nicht-interventionell: **[---]***
- Studiendesign Zuteilung: **Kontrollierte, randomisierte Studie**
- Verblindung: **Offen**

Studientyp: **Interventionell**

Studientyp nicht-interventionell: **[---]***

Studiendesign Zuteilung: **Kontrollierte, randomisierte Studie**

Verblindung: **Offen**

- Wer ist verblindet: **[---]***
- Kontrolle: **Aktive Kontrolle**
- Studienzweck: **Therapie**
- Gruppenzuteilung: **Parallelverteilung**
- Studienphase: **IIIb**
- Off-label Drug use: **Nein**

Primärer Endpunkt

Rate der Abheilung der Anastomoseninsuffizienz (radiologisch oder endoskopisch) bzw. Krankenhaus-Entlassfähigkeit nach 44 Tagen.

Sekundärer Endpunkt

1. Krankenhausverweildauer
2. Rate an Therapieversagern (definiert durch einen notwendige Änderung der Behandlungsstrategie inkl. operativer Revision)
3. Notwendigkeit einer operativen Revision und deren Art
4. Komplikationsraten allgemein und spezifisch für die angewandte Therapie (z.B. Stent-Dislokationen)
5. Sterblichkeit
6. Intensivbehandlungszeit
7. Einfluss der Behandlung auf eine mögliche Sepsis gemessen anhand des SOFA- Scores
8. Beatmungsdauer
9. Lebensqualität (EORTC QLQ C30, EORTC QLQ – OG25 Fragebogen) nach 3 Monaten
10. Dauer der jeweiligen Therapie
11. Dauer bis zur endoskopisch verifizierten Heilung
12. Dauer zwischen Diagnose und Kostaufbaus peroral
13. Behandlungskosten
14. Auftreten von symptomatischen Stenosen

Länder in denen Studienteilnehmer rekrutiert werden

- DE **Deutschland**

Rekrutierungsstandorte

- Universitätsklinikum **Hamburg**

Rekrutierung

- Geplant/Tatsächlich: **Geplant**
- (geplantes/tatsächliches Datum) Einschluss des ersten Studienteilnehmers: **17.04.2015**
- Geplante Studienteilnehmeranzahl gesamt: **78**
- Monozentrisch/Multizentrisch: **Monozentrisch**
- National/International: **National**

Einschlusskriterien

- Geschlecht: **Beide, männlich und weiblich**
- Mindestalter: **18 Jahre**
- Höchstalter: **99 Jahre**

Weitere Einschlusskriterien

1. Histologisch bestätigtes Ösophagus- oder Magenkarzinom oder in ähnlicher Weise operierte Neoplasie (z.B. GIST)
2. Ösophagektomie oder Gastrektomie mit einer intrathorakalen oder abdominellen ösophago-enterischen Anastomose
3. Radiologisch oder endoskopisch diagnostizierte ösophago-enterische Anastomoseninsuffizienz
4. Klinische Beschwerden/ Symptome aufgrund der Insuffizienz oder Erhöhung der Entzündungszeichen, am ehesten als Folge der Anastomoseninsuffizienz
5. Alter ≥18 Jahre
6. Befähigung des Patienten, den Studienumfang und seine Konsequenzen bzw. die Aufklärung darüber zu verstehen, darin einzuwilligen und die Aufklärungsunterlagen zu unterschreiben.
7. Schriftliche Einwilligungserklärung des einzuschließenden Patienten. Ist der Patient nicht in der Lage, eigenhändig zu unterschreiben, muss ein Zeuge die erfolgte mündliche Aufklärung durch Unterschrift bestätigen.

Ausschlusskriterien

1. Makroskopisch unvollständig resezierter Tumor (R2), Palliativresektion
2. Endoskopisch verifizierte Nekrose des Interponates
3. Größe der Insuffizienz > 2/3 Zirkumferenz
4. Frühe Anastomoseninsuffizienz (≤ 48 Stunden postoperativ)
5. Schwangere und stillende Frauen

Adressen

- **Primärer Sponsor**

  Universitätsklinikum Hamburg-Eppendorf
  Martinistr. 52
  20246 Hamburg
  Deutschland

  Telefon: **[---]***
  Fax: **[---]***
  E-Mail: **[---]***
  URL der Einrichtung: **www.uke.de**

- **Kontakt für wissenschaftliche Anfragen**

  Universitätsklinikum Hamburg-Eppendorf
  Herr Dr. med. Michael Tachezy
  Martinistr 52
  20146 Hamburg
  Deutschland

  Telefon: **+4940741052401**
  Fax: **[---]***
  E-Mail: **mtachezy at uke.de**
  URL der Einrichtung: **www.uke.de**

- **Kontakt für Studienteilnehmer**

  Universitätsklinikum Hamburg-Eppendorf
  Herr Dr. med. Michael Tachezy
  Martinistr 52
  20146 Hamburg
  Deutschland

  Telefon: **+4940741052401**
  Fax: **[---]***
  E-Mail: **mtachezy at uke.de**
  URL der Einrichtung: **www.uke.de**

- **Kooperationspartner, sonstige Adresse**

  Universitätsklinikum Hamburg-Eppendorf
  Herr Dr. med. Stefan Groth
  Martinistr 52
  20246 Hamburg
  Deutschland

  Telefon: **[---]***
  Fax: **[---]***

Kooperationspartner, sonstige Adresse

Universitätsklinikum Hamburg-Eppendorf
Herr Dr. med. Stefan Groth
Martinistr 52
20246 Hamburg
Deutschland

Telefon: **[---]***
Fax: **[---]***
E-Mail: **groth at uke.de**
URL der Einrichtung: **www.uke.de**

Finanzierungsquellen

- **Private Gelder (Stiftungen, Studiengesellschaften etc.)**

Hamburger Krebsgesellschaft e.V.
Butenfeld 18
22528 Hamburg
Deutschland

Telefon: **[---]***
Fax: **[---]***
E-Mail: **[---]***
URL der Einrichtung: **[---]***

- **Haushaltsmittel, keine fremden Finanzmittel (Budget des Studienleiters)**

Universitätsklinikum Hamburg-EppendorfKlinik für Allgemein, Viszeral und ThoraxchiurgieKlinik für Interdisziplinäre Endoskopie
Martinistr 52
20246 Hamburg
Deutschland

Telefon: **[---]***
Fax: **[---]***
E-Mail: **[---]***
URL der Einrichtung: **[---]***

Status

- Status der Rekrutierung: **Rekrutierung läuft**
- Tatsächliches Datum des Studienabschlusses (LPLV): **[---]***

Publikationen, Studienergebnisse und weitere Studiendokumente

** Dieser Eintrag bedeutet, dass der Parameter entweder nicht zutrifft oder dass er nicht eingetragen wurde.*

**** Dieser Eintrag bedeutet, dass Daten aus datenschutzrechlichen Gründen nicht angezeigt werden.*
